# Soluble urokinase plasminogen activation receptor and long-term outcomes in persons undergoing coronary angiography

**DOI:** 10.1038/s41598-018-36960-6

**Published:** 2019-01-24

**Authors:** Claudia Sommerer, Martin Zeier, Christian Morath, Jochen Reiser, Hubert Scharnagl, Tatjana Stojakovic, Graciela E. Delgado, Winfried März, Marcus E. Kleber

**Affiliations:** 1 Department of Nephrology, University of Heidelberg, Heidelberg, Germany; 2 Department of Medicine, Rush University Medical Center, 1735 West Congress Parkway, Suite 1004, Chicago, IL 60612 USA; 3 Clinical Institute of Medical and Chemical Laboratory Diagnostics, Medical University of Graz, Graz, Austria; 4 Vth Department of Medicine, Medical Faculty Mannheim of the University of Heidelberg, Mannheim, Germany; 5 Synlab Academy, Synlab Holding Deutschland GmbH, Mannheim, Germany

## Abstract

Soluble urokinase plasminogen activation receptor (suPAR) is risk factor for kidney disease and biomarker for cardiovascular outcomes but long term longitudinal analyses in a large European cohort have not been perfomed. To hus, we studied suPAR in participants of the Ludwigshafen Risk and Cardiovascular Health study over a very long follow-up time of nearly 10 years. We estimated overall risk of all-cause and cardiovascular death by Cox proportional hazards regression according to quartiles of suPAR, including age, sex, use of lipid-lowering drugs, body mass index, diabetes mellitus, hypertension, smoking, lipids, as well as glomerular filtration rate (eGFR), NT-proBNP, interleukin-6 and high-sensitive CRP as covariates. A total of 2940 participants (age 62.7 ± 10.5years) having a median eGFR of 83.8 mL/min/1.73 m^2^ were included. The median suPAR concentration was 3010 pg/mL (interquartile range, 2250–3988 pg/mL). Using the lowest quartile of suPAR as the reference, crude hazard ratio for cardiovascular mortality were 1.58 (95% CI 1.16–2.16), 1.85 (95% CI 1.37–2.52) and 2.75 (95% CI 2.03–3.71) in the second, third and fourth quartile, respectively. Adjusting for NT-proBNPeGFR or inflammation (interleukin-6 and high-sensitive CRP) confirmed results. suPAR predicts all-cause and cardiovascular death over a period of ten years in persons undergoing coronary angiography, independent of the natriuretic peptide NT-proBNP, kidney function and of markers of systemic inflammation. Future investigation into a potential causal role of suPAR in cardiovascular disease is warranted.

## Introduction

Cardiovascular events are still the main causes of death around the world. Predicting and managing cardiovascular risk is key for appropriate resourcing of health care and managing diseases burden. In this regard, soluble urokinase plasminogen activator receptor (suPAR), a signaling molecule and emerging biomarker of prognostic value in kidney disease and other settings, including cardiovascular (CV) disease^1,2^ has been gaining much attention. Recently, suPAR was suggested to be directly involved in a pathophysiological pathway closely linked to atherosclerosis^3,4^. Urokinase-type plasminogen activator receptor is a membrane-linked protein resident in several cell types, e.g. immunologically active cells and endothelial cells^4,5^. This is an important receptor in many physiological pathways such as cell signaling, modulation of cell adhesion, migration, and proliferation. The soluble form of the urokinase-type plasminogen activator receptor, suPAR, may also be a scavenger of vitronectin, a plasma glycoprotein that has been implicated in coronary atherosclerosis^4^. Further, suPAR has been linked to neointimal formation of atheroscerlotic lesions^3^. In clinical investigations, suPAR has been predictive of de-novo kidney disease and has also been linked to other disease conditions such as coronary artery disease (CAD), ischemic stroke, diabetes mellitus, infection, sepsis as well as malignancy^6,7^. suPAR is related to inflammation, but presents unique features different from other inflammatory biomarkers. While the pathophysiology of suPAR in renal disease involves podocyte integrin activation^8^, a causal role for suPAR in cardiac disease is speculative. suPAR levels have been shown to predict CV events and chronic kidney disease (CKD)^7,9–11^.

The present study represents a large analysis of the cardio-renal biomarker suPAR as predictor of mortality in an extensively characterized unselected European Caucasian population with a very long follow-up time of nearly 10 years. Using a baseline measurement of suPAR in the Ludwigshafen Risk and Cardiovascular Health (LURIC) study cohort, all relevant traditional and more recently established cardiovascular risk factors, we characterize the association between suPAR and all-cause as well as CV death. In addition, the additional value of suPAR to all traditional and well-known CV risk factors is evaluated by adjusting for these strong predictors of CV mortality and diseases.

## Results

### Patient population

Baseline suPAR assessments were available in 2940 of 3316 (88.7%) patients enrolled in the LURIC study. Patients were 68.4% (2012/2940) men; the mean age was 62.8 ± 10.5years. Median estimated glomerular filtration rate (eGFR) was 83.8 mL/min/1.73 m^2^ with 398 patients showing an eGFR <60 mL/min/1.73 m^2^. The median follow-up period was 9.9years (0.1–11.9). Detailed patients´ characteristics are presented in Table 1.

**Table 1:** Baseline population characteristics per quartile of soluble urokinase plasminogen activator receptor (suPAR).

Characteristics	all	suPAR Quartile 1	suPAR Quartile 2	suPAR Quartile 3	suPAR Quartile 4	p*
suPAR (pg/mL)		<2240	2250–3000	3010–3980	≥3990	
N =	2940	729	740	736	735	
Median Follow-up (years)	9.89 (8.59–10.7)	10.2 (9.63–10.8)	10.0 (9.47–10.7)	9.91 (8.02–10.7)	9.47 (4.49–10.4)	<0.001
Age (years)	62.8 ± 10.5	59.3 ± 10.1	61.8 ± 10.2	64.3 ± 9.80	65.6 ± 10.6	<0.001
Gender, male (%)	68.4	76.1	70.8	63.3	63.5	<0.001
Body mass index (kg/m^2^)	27.5 ± 4.10	27.4 ± 3.77	27.4 ± 3.76	27.6 ± 4.13	27.6 ± 4.70	0.680
S-creatinine (µmol/L)	86.6 ± 35.4	80.4 ± 14.1	81.3 ± 15.9	83.1 ± 17.7	99.9 ± 63.6	<0.001
eGFR, CKDepi (mL/min)	81.7 ± 20.1	91.1 ± 14.7	86.3 ± 16.6	81.0 ± 17.7	68.3 ± 22.8	<0.001
eGFR, MDRD (mL/min)	81.2 ± 19.0	87.1 ± 15.9	84.4 ± 16.9	80.7 ± 17.2	72.5 ± 22.3	<0.001
Systolic blood pressure (mmHg)	141 ± 23.7	139 ± 21.8	141 ± 22.6	144 ± 23.8	142 ± 26.1	<0.001
Diastolic blood pressure (mmHg)	81.0 ± 11.4	81.5 ± 11.2	81.3 ± 11.2	81.8 ± 11.1	79.2 ± 11.8	<0.001
Mean arterial blood pressure (mmHg)	111 ± 14.8	110 ± 14.6	111 ± 14.5	113 ± 14.6	111 ± 15.3	0.003
Pulse pressure	64.7 ± 18.5	61.3 ± 16.4	63.9 ± 17.6	66.6 ± 18.5	67.1 ± 20.7	<0.001
History of coronary artery disease (%)	78.3	70.2	77.3	81.0	84.6	<0.001
Gensini Score	31.5 (7.00–64.0)	23.5 (1.00–58.0)	30.5 (5.50–60.9)	32.0 (10.0–62.0)	37.5 (15.0–73.0)	<0.001
0–1–2–3 vessel disease** (%)	32.0/18.9/19.1/30.0	39.1/18,8/18.8/23.3	33.8/19.5/17.0/29.7	28.9/19.8/21.9/29.3	26.3/17.4/18.9/37.4	<0.001
History of congestive heart failure (%)	32.9	23.0	28.6	35.7	43.9	<0.001
History of peripheral vascular disease (%)	9.2	5.3	7.8	9.9	13.7	<0.001
History of stroke (%)	9.3	5.1	8.1	11.1	12.8	<0.001
History of myocardial infarction (%)	41.5	35.4	36.4	43.8	50.5	<0.001
Hypertension (%)	72.9	67.2	71.9	76.5	75.8	<0.001
Diabetes mellitus (%)	39.6	26.6	34.7	42.4	54.7	<0.001
Statin treatment (%)	47.7	45.5	45.5	50.1	49.5	0.140
Smoker (%)	23.0	17.6	23.5	23.2	27.6	<0.001
Ex-smoker (%)	41.0	42.9	42.0	38.5	40.5	<0.001
Hemoglobin (mmol/L)	8.57 ± 0.92	8.75 ± 0.74	8.63 ± 0.88	8.56 ± 0.89	8.25 ± 1.0.8	<0.001
Albumin (g/L)	439 ± 53	453 ± 53	444 ± 53	438 ± 52	423 ± 57	<0.001
HbA1c (mmol/mol)	45 ± 8.68	42.1 ± 6.37	44.9 ± 8.33	46.5 ± 9.52	48.6 ± 9.66	<0.001
LDL-C (mmol/L)	3.02 ± 0.89	3.07 ± 0.86	3.02 ± 0.89	3.04 ± 0.89	2.96 ± 0.92	0.080
HDL-C (mmol/L)	1.01 ± 0.28	1.07 ± 0.28	1.03 ± 0.27	1.02 ± 0.29	0.93 ± 0.27	<0.001
Triglycerides (mmol/L)	1.66 (1.23–2.27)	1.66 (1.22–2.25)	1.67 (1.21–2.29)	1.63 (1.21–2.25)	1.64 (1.26–2.22)	0.818
Calcium (mmol/L)	2.33 ± 0.11	2.33 ± 0.10	2.33 ± 0.11	2.33 ± 0.11	2.32 ± 0.12	0.279
Phosphate (mmol/L)	1.14 ± 0.18	1.11 ± 0.16	1.14 ± 0.18	1.13 ± 0.18	1.18 ± 0.21	<0.001
Intact parathormone (pmol/L)	3.08 (2.33–4.14)	2.97 (2.23–3.82)	3.08 (2.23–3.92)	3.08 (2.23–4.14)	3.39 (2.44–4.98)	<0.001
White blood cells (/nL)	7.08 ± 2.12	6.74 ± 1.82	6.99 ± 2.03	7.15 ± 2.11	7.43 ± 2.42	<0.001
hsCRP (mg/L)	3.42 (1.31–8.73)	1.90 (0.89–4.92)	2.80 (1.15–7.33)	3.74 (1.56–8.75)	6.52 (2.66–17.2)	<0.001
IL-6 (ng/L)	3.21 (1.79–6.08)	2.38 (1.45–4.21)	2.80 (1.67–4.79)	3.35 (1.84–6.37)	5.04 (2.46–9.82)	<0.001
NT-pro BNP (pmol/L)	35.3 (12.7–104.8)	18.6 (8.85–45.9)	30.4 (10.4–77.5)	36.1 (15.3–96.2)	92.6 (29.9–259.8)	<0.001
Use of ACE-I (%)	53.2	44.3	51.5	54.8	62.3	<0.001
Use of ARB (%)	4.5	4.3	3.0	5.4	5.3	<0.001
Use of Calciumantagonists (%)	15.7	14.7	14.3	16.4	17.3	0.344
Use of β-blocker (%)	64.1	62.8	63.8	67.1	62.7	0.254
Use of diuretics (%)	28.7	18.5	22.3	27.0	46.8	<0.001
Antiplatelet drug (%)	71.8	70.1	71.4	74.7	70.9	0.209
Anticoagulation (%)	6.8	4.5	5.8	6.7	10.1	<0.001

mean ± SD or median (25th-75th percentile) *ANOVA for continuous variables (non-normally distributed variables were log-transformed before entering analysis) or χ^2^ test for categorial variables, **CAD defined as at least one stenosis ≥50%.

ACE-I, angiotensin converting enzyme inhibitor; ARB, angiotensin receptor blocker; CKDepi, epidemiological chronic kidney disease (formula); eGFR, estimated glomerular filtration rate; HbA1c, glycosylated hemoglobin; HDL-C, high density lipoprotein cholesterol; hsCRP, high sensitivity C-reactive protein; IL-6, interleukin 6; LDL-C, low density lipoprotein cholesterol; MDRD, modification of diet in renal disease (formula); NT-proBNP, N- terminal pro brain natriuretic peptide; N, number; p, significance; S-creatinine, serum creatinine.

### suPAR analysis

Median suPAR level was 3010 pg/mL (interquartile range, 2250–3988 pg/mL). Patients with high suPAR concentrations were more likely to be female, smokers, and more frequently had the diagnosis of hypertension, diabetes mellitus, congestive heart failure, and CV disease as compared to patients with low suPAR concentrations (Table 1). High suPAR levels were associated with higher age and blood pressure, higher concentrations of phosphate, iPTH and HbA1c as well as lower albumin, hemoglobin, and HDL-C.

Mean NT-pro BNP increased with each suPAR quartile. In addition, patients with high suPAR levels presented increased inflammation markers as leukocytes, hs-CRP and IL-6. Patients with high suPAR levels were more likely to have renal impairment. suPAR levels were correlated inversely with eGFR.

suPAR levels were significantly associated with prevalent CAD at baseline, heart failure, and CKD adjusted for age and sex as well as after additional adjustment for traditional risk factors as body-mass index (BMI), hypertension, smoking, and hyperlipidemia. Odds ratio for CAD, heart failure and diabetes mellitus increased significantly per one standard deviation increase of suPAR levels. Especially, the prevalence of CKD rose with suPAR levels (Table 2).

**Table 2:** Prevalence (odds ratio and 95% confidence of coronary artery disease, heart failure, diabetes mellitus and chronic kidney disease per one standard deviation increase in soluble urokinase plasminogen activator receptor (suPAR) concentration (n = 2940).

	Odds ratio (95% CI) per 1 SD increase in suPAR			
	Model 1 Demographics	p	Model 2 Demographics + traditional CV risk factors	p
	OR (95% CI)		OR (95% CI)	
**Coronary artery disease**				
1st	1		1	
2nd	1.43 (1.12–1.86)	0.004	1.31 (1.00–1.73)	0.051
3rd	1.78 (1.37–2.31)	<0.001	1.47 (1.10–1.97)	0.009
4th	2.25 (1.70–2.96)	<0.001	1.63 (1.20–2.22)	0.002
**Heart failure**				
1st	1		1	
2nd	1.26 (0.99–1.60)	0.060	1.19 (0.93–1.51)	0.164
3rd	1.66 (1.31–2.10)	<0.001	1.54 (1.21–1.96)	<0.001
4th	2.25 (1.78–2.84)	<0.001	1.90 (1.49–2.43)	<0.001
**Diabetes mellitus**				
1st	1		1	
2nd	1.36 (1.08–1.70)	0.009	1.28 (1.01–1.62)	0.039
3rd	1.75 (1.40–2.19)	<0.001	1.60 (1.27–2.03)	<0.001
4th	2.77 (2.21–3.48)	<0.001	2.38 (1.87–3.03)	<0.001
**Chronic kidney disease**				
1st	1		1	
2nd	1.62 (0.92–2.85)	0.096	1.55 (0.88–2.75)	0.132
3rd	3.23 (1.92–5.43)	<0.001	3.11 (1.84–5.25)	<0.001
4th	12.9 (7.88–21.02)	<0.001	12.41 (7.51–20.51)	<0.001

CI, confidence interval; OR, odds ratio; p, significance; SD, standard deviation.

Model 1: adjusted for age and sex.

Model 2: additionally adjusted for lipid lowering therapy, BMI, hypertension, smoking, LDL-C, HDL-C and logarithmic triglyceride.

In the multivariate regression analysis, suPAR was independently influenced by eGFR, HDL-C, albumin, hemoglobin, blood pressure, yGT, IL-6, HbA1c, NT-proBNP, smoking status, and female gender (Table 3).

**Table 3:** Multivariate regression analysis of soluble urokinase plasminogen activator receptor (suPAR).

Variable	Estimate	Std. Error	t value	Pr (>|t|)
Age	−0.05587	0.03416	−1.636	0.102
eGFR	−0.55601	0.03344	−16.625	<0.001
Albumin	−0.12963	0.02681	−4.835	<0.001
Hemoglobin	−0.08801	0.0303	−2.904	0.004
White blood cells	0.04185	0.02715	1.541	0.123
hsCRP	0.02155	0.03093	0.697	0.486
IL-6	0.10809	0.03079	3.511	<0.001
HDL-C	−0.18904	0.0271	−6.976	<0.001
yGT	0.20957	0.02627	7.978	<0.001
phosphate	0.02766	0.02697	1.026	0.305
Intact parathormone	0.04399	0.03069	1.434	0.152
Hba1c	0.11282	0.02574	4.383	<0.001
NT-proBNP	0.17186	0.02898	5.931	<0.001
Diastolic blood pressure	−0.07166	0.03501	−2.047	0.041
Systolic blood pressure	0.08342	0.03663	2.277	0.023
Female sex	0.25851	0.06638	3.895	<0.001
Ex-smoker	0.23783	0.06115	3.889	<0.001
Active smoker	0.48327	0.07403	6.528	<0.001

All variables were Z-transformed before analysis.

eGFR, estimated glomerular filtration rate; yGT, gamma glutamyltransferase; HbA1c, glycosylated hemoglobin; HDL-C, high density lipoprotein cholesterol; hsCRP, high sensitivity C-reactive protein; IL-6, interleukin 6; NT-proBNP, N- terminal pro brain natriuretic peptide.

### suPAR and fatal outcomes

#### All-cause mortality

**Figure 1:**
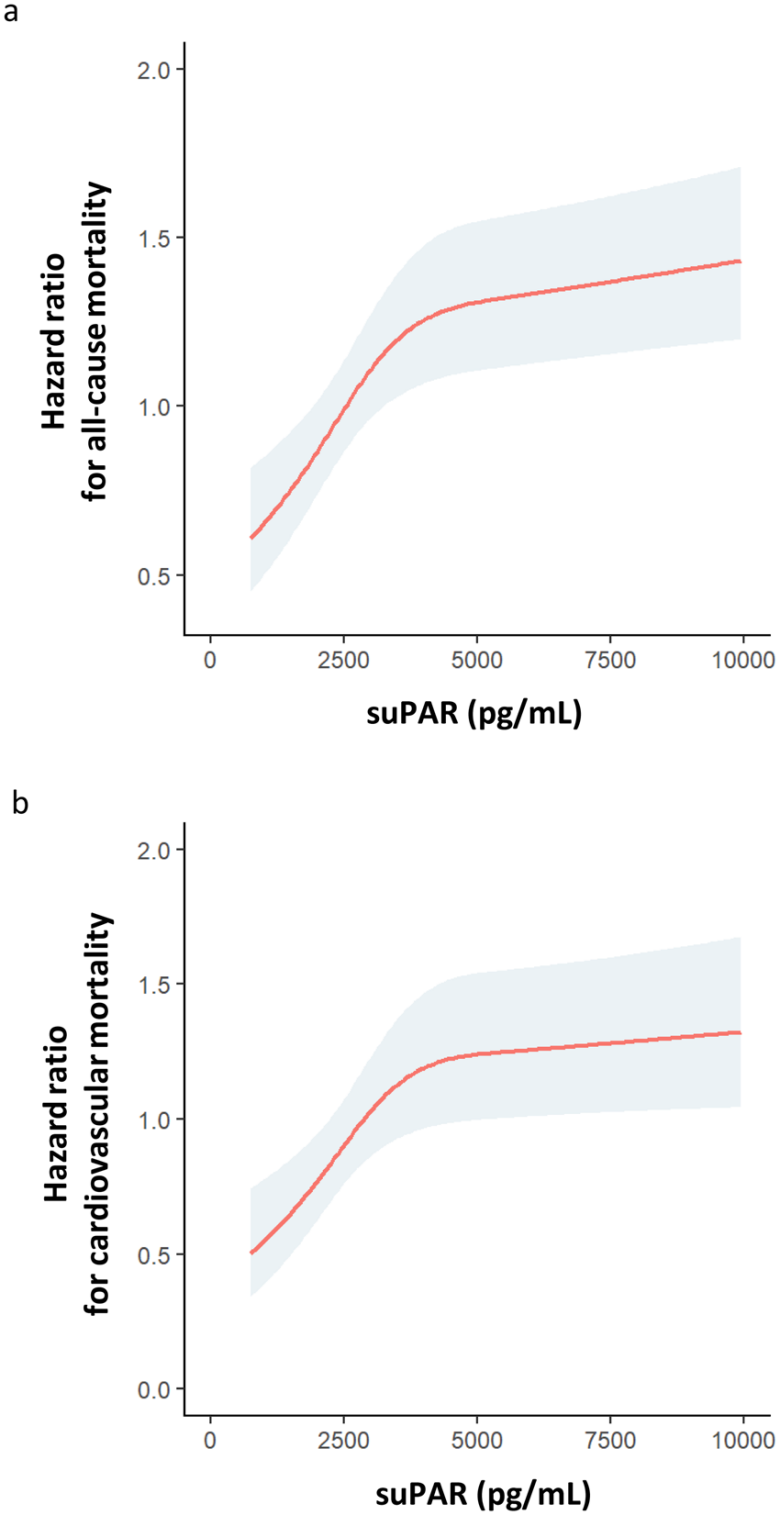
Risk (hazard ratio and 95% confidence interval) of a. all-cause mortality and b. cardiovascular mortality depending on soluble urokinase plasminogen activator receptor (suPAR) concentration (pg/mL) at baseline (study population n = 2940).

**Figure 2:**
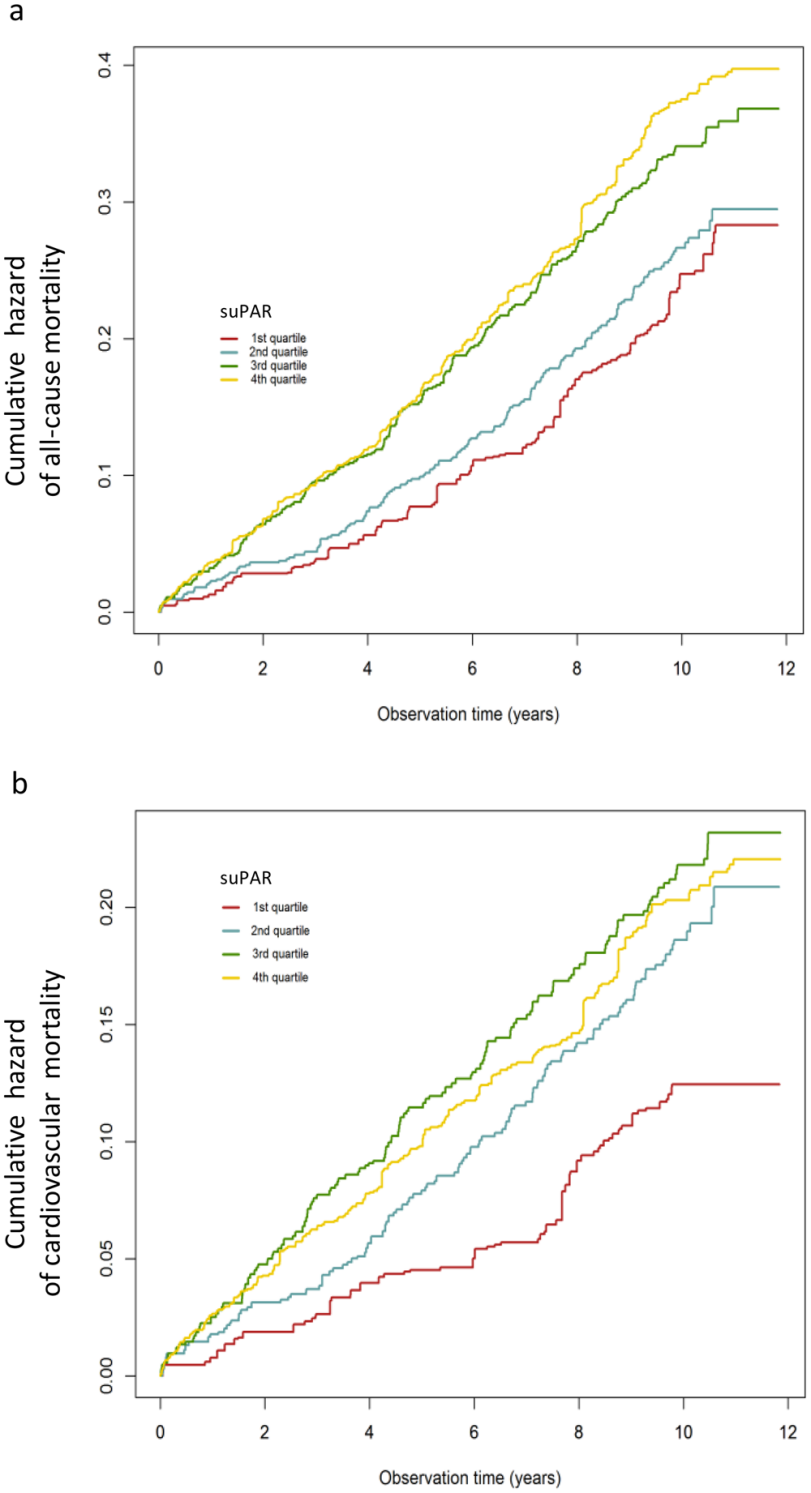
Event-free survival stratified by soluble urokinase plasminogen activator receptor (suPAR) quartiles. Kaplan-Meier curves for the time to a. all-cause mortality and b. cardiovascular mortality.

During the follow-up period of 9.9years 873 (28.5%) patients died. High suPAR levels at baseline were associated with an increased risk for all-cause death (Fig. 1a). The patient cohort was divided into quartiles according to their suPAR levels. Event-free survival decreased with suPAR quartile (Fig. 2a). The unadjusted risk was incrementally higher with rising suPAR quartile. Patients in higher suPAR quartiles exhibited higher mortality risk after adjustment for age and sex (second quartile: HR 1.26; 95% CI, 1.0 to 1.6; third quartile: HR 1.69; 95% CI, 1.35–2.11; fourth quartile: HR 1.86; 95% CI, 2.31–3.52). This association remained significant after adjustment for additional confounders including strong biomarkers such as NT-proBNP, IL-6, and CRP (Table 4).

**Table 4:** Risk (hazard ratio and 95% confidence interval) of all-cause mortality and cardiovascular mortality stratified by quartiles of soluble urokinase plasminogen activator receptor (suPAR) concentration (pg/mL) at baseline (study population n = 2940).

	Model 1 Demographics		Model 2 Demographics + traditional CV risk factors		Model 3 Demographics + traditional CV risk factors + specific CV risk factors	
	HR (95% CI)	p	HR (95% CI)	p	HR (95% CI)	p
**All-cause mortality (873 events)**						
Quartile 1	1 (reference)		1 (reference)		1 (reference)	
Quartile 2	1.26 (1.00–1.60)	0,051	1.18 (0.94–1.50)	0.158	1.12 (0.88–1.43)	0.344
Quartile 3	1.69 (1.35–2.11)	<0.001	1.51 (1.21–1.90)	<0.001	1.31 (1.04–1.66)	0.022
Quartile 4	2.86 (2.31–3.53)	<0.001	2.36 (1.89–2.94)	<0.001	1.65 (1.30–2.09)	<0.001
**Cardiovascular mortality (538 events)**						
Quartile 1	1 (reference)		1 (reference)		1 (reference)	
Quartile 2	1.68 (1.23–2.30)	0.001	1.58 (1.16–2.16)	0.004	1.52 (1.11–2.09)	0.01
Quartile 3	2.10 (1.55–2.85)	<0.001	1.85 (1.37–2.52)	<0.001	1.56 (1.14–2.15)	0.006
Quartile 4	3.43 (2.56–4.58)	<0.001	2.75 (2.03–3.71)	<0.001	1.78 (1.28–2.46)	<0.001

mean ± SD or median (25th-75th percentile).

Model 1: adjusted for age and sex.

Model 2: additionally adjusted for lipid lowering therapy, coronary artery disease status, body-mass index, diabetes mellitus, hypertension, smoking, LDL-C, HDL-C and log triglycerides.

Model 3: additionally adjusted for estimated glomerular filtration rate, NT-proBNP, interleukin 6 and high-sensitive C-reactive protein.

CI, confidence interval; HR, hazard ratio; p, significance.

#### Cardiovascular mortality

In 538 patients (18.3%) death was caused by CV events. High suPAR levels at study baseline were associated with an increased risk CV death in the follow-up period (Fig. 1b). The patient cohort was divided into quartiles according to their suPAR levels. Event-free survival decreased with suPAR quartile (Fig. 2b). The unadjusted risk was incrementally higher with rising suPAR quartile (second quartile: HR 1.68; 95% CI 1.23–2.30; third quartile: HR 1.10; 95% CI 1.55–2.85; fourth quartile: HR 2.43; 95% CI 2.56–4.58). This association remained significant after adjustment for additional confounders (Table 4), again including biomarkers.

As shown in Table 5, the risk of all components of the endpoint of CV death increased with each quartile of suPAR levels. When we analyzed suPAR as a continuous variable, a 42% higher CV mortality risk was found per doubling of the suPAR concentration.

**Table 5:** Risk (hazard ratio and 95% confidence interval) of sudden cardiac death, fatal myocardial infarction, fatal stroke, death due to congestive heart failure stratified by quartiles of soluble urokinase plasminogen activator receptor (suPAR) concentration (pg/mL) at baseline (study population n = 2940).

	Model 1 Demographics		Model 2 Demographics + traditional CV risk factors		Model 3 Demographics + traditional CV risk factors + specific CV risk factors	
	HR (95% CI)	p	HR (95% CI)	p	HR (95% CI)	p
**Sudden cardiac death (220 events)**						
Quartile 1	1 (reference)		1 (reference)		1 (reference)	
Quartile 2	1.70 (1.04–2.80)	0.036	1.67 (1.01–2.75)	0.044	1.52 (0.92–2.50)	0.102
Quartile 3	2.03 (1.24–3.30)	0.005	1.91 (1.17–3.13)	0.010	1.52 (0.93–2.51)	0.098
Quartile 4	4.11 (2.61–6.49)	<0.001	3.74 (2.33–5.99)	<0.001	2.27 (1.38–3.74)	0.001
**Fatal myocardial infarction (90 events)**						
Quartile 1	1 (reference)		1 (reference)		1 (reference)	
Quartile 2	1.62 (0.77–3.41)	0.205	1.39 (0.66–2.94)	0.384	1.57 (0.71–3.49)	0.270
Quartile 3	2.78 (1.39–5.58)	0.004	2.11 (1.04–4.28)	0.037	2.13 (0.98–4.63)	0.055
Quartile 4	2.84 (1.40–5.77)	0.004	1.76 (0.84–3.67)	0.133	1.48 (0.64–3.43)	0.360
**Fatal stroke (55 events)**						
Quartile 1	1 (reference)		1 (reference)		1 (reference)	
Quartile 2	3.35 (1.12–10.0)	0.031	3.06 (1.02–9.20)	0.047	3.02 (1.00–9.09)	0.049
Quartile 3	3.41 (1.14–10.2)	0.028	2.85 (0.94–8.64)	0.064	2.60 (0.85–7.97)	0.095
Quartile 4	3.57 (1.18–10.8)	0.024	2.50 (0.80–7.81)	0.116	2.09 (0.64–6.84)	0.225
**Death due to congestive heart failure (130 events)**						
Quartile 1	1 (reference)		1 (reference)		1 (reference)	
Quartile 2	1.56 (0.84–2.88)	0.156	1.43 (0.78–2.65)	0.250	1.36 (0.72–2.55)	0.339
Quartile 3	1.55 (0.84–2.86)	0.163	1.33 (0.71–2.48)	0.370	1.05 (0.55–2.01)	0.878
Quartile 4	3.06 (1.73–5.42)	<0.001	2.35 (1.30–4.26)	0.005	1.28 (0.67–2.45)	0.458

mean ± SD or median (25th-75th percentile).

Model 1: adjusted for age and sex.

Model 2: additionally adjusted for lipid lowering therapy, coronary artery disease status, body-mass index, diabetes mellitus, hypertension, smoking, LDL-C, HDL-C and log triglycerides.

Model 3: additionally adjusted for estimated glomerular filtration rate, NT-proBNP, interleukin 6 and high-sensitive C-reactive protein.

CI, confidence interval; HR, hazard ratio; p, significance.

#### Death from infections

In 72 patients (2.4%) death was caused by infectious events. High suPAR levels at study baseline were associated with an increased risk to die due to fatal infection. When the patient cohort was divided into quartiles according to their suPAR levels, the unadjusted risk was incrementally higher with rising suPAR quartile (Table S1). This association remained significant after adjustment for confounders.

Analyzing suPAR as continuous variable, a 64% higher risk for fatal infection was found per doubling in suPAR concentrations (1.64, 1.08–2.47).

### suPAR for risk assessment compared in addition to traditional risk factors

We examined whether adding suPAR to traditional CV risk factors as age, sex, body-mass index, LDL-C, HDL-C, smoking, hypertension, diabetes mellitus improved risk stratification for both, all-cause and CV death improved risk stratification (Table 6). Addition of suPAR to a model with traditional risk factors significantly improved the prediction of all-cause death and CV death (all-cause death, delta 0.016, p < 0.001; CV death, delta 0.005, p < 0.001). Addition of suPAR to a model with traditional risk factors also improved significantly risk stratification in the CAD subgroup.

**Table 6:** Prediction of all-cause mortality and cardiovascular mortality with traditional risk factors (age, sex, body-mass index, LDL-C, HDL-C, smoking, hypertension, diabetes mellitus) as well as heart failure and with and without soluble urokinase plasminogen activator receptor (suPAR) concentration.

All-cause mortality			
	Harrells C	AUC (95% CI)	P
**All participants (n = 2940)**			
Base	0.717	0.755 (0.736–0.774)	
Base + suPAR	0.723	0.771 (0.753–0.790)	<0.001*
Base + hsCRP	0.718	0.755 (0.737–0.774)	0.435*
Base + IL-6	0.724	0.762 (0.743–0.780)	0.002*
Base + NT-proBNP	0.731	0.777 (0.759–0.795)	<0.001*
Base + NT-proBNP + suPAR	0.734	0.785 (0.767–0.802)	<0.001**
**Only CAD patients (n = 2302)**			
Base	0.702	0.742 (0.720–0.763)	
Base + suPAR	0.707	0.757 (0.737–0.778)	<0.001*
Base + hsCRP	0.702	0.742 (0.721–0.763)	0.779*
Base + IL-6	0.708	0.747 (0.726–0.767)	0.017*
Base + NT-proBNP	0.716	0.764 (0.743–0.784)	<0.001*
Base + NT-proBNP + suPAR	0.719	0.770 (0.750–0.790)	<0.001**
**Only CKD patients (n = 398)**			
Base	0.606	0.670 (0.617–0.723)	
Base + suPAR	0.641	0.706 (0.654–0.758)	0.035*
Base + hsCRP	0.606	0.673 (0.620–0.730)	0.571*
Base + IL-6	0.610	0.671 (0.618–0.724)	0.859*
Base + NT-proBNP	0.630	0.688 (0.635–0.740)	0.073*
Base + NT-proBNP + suPAR	0.650	0.708 (0.656–0.759)	0.184**
**cardiovascular mortality**			
	**Harrells C**	**AUC (95% CI)**	**p**
**All participants (n = 2922)**			
Base	0.721	0.727 (0.705–0.749)	
Base + suPAR	0.726	0.738 (0.716–0.760)	<0.001*
Base + hsCRP	0.722	0.728 (0.706–0.750)	0.328*
Base + IL-6	0.728	0.734 (0.711–0.756)	0.004*
Base + NT-proBNP	0.738	0.752 (0.731–0.774)	<0.001*
Base + NT-proBNP + suPAR	0.742	0.755 (0.734–0.777)	0.003**
**Only CAD patients (n = 2284)**			
Base	0.700	0.704 (0.679–0.729)	
Base + suPAR	0.706	0.715 (0.690–0.740)	<0.001*
Base + hsCRP	0.701	0.704 (0.679–0.730)	0.053*
Base + IL-6	0.707	0.710 (0.685–0.735)	0.028*
Base + NT-proBNP	0.718	0.730 (0.706–0.755)	<0.001*
Base + NT-proBNP + suPAR	0.722	0.733 (0.709–0.757)	0.024**
**Only CKD patients (n = 393)**			
Base	0.612	0.616 (0.560–0.673)	
Base + suPAR	0.645	0.633 (0.577–0.688)	0.081*
Base + hsCRP	0.612	0.620 (0.564–0.676)	0.348*
Base + IL-6	0.618	0.620 (0.564–0.676)	0.372*
Base + NT-proBNP	0.636	0.626 (0.570–0.682)	0.298*
Base + NT-proBNP + suPAR	0.654	0.635 (0.579–0.690)	0.215**

*P versus base model; **P versus Base + NT-proBNP.

AUC, area under the curve; CAD, coronary artery disease; CI, confidence interval; CKD, chronic kidney disease; p significance; suPAR, soluble urokinase plasminogen activator receptor.

### SuPAR and mortality subgroups

#### Patients with coronary artery disease

**Figure 3:**
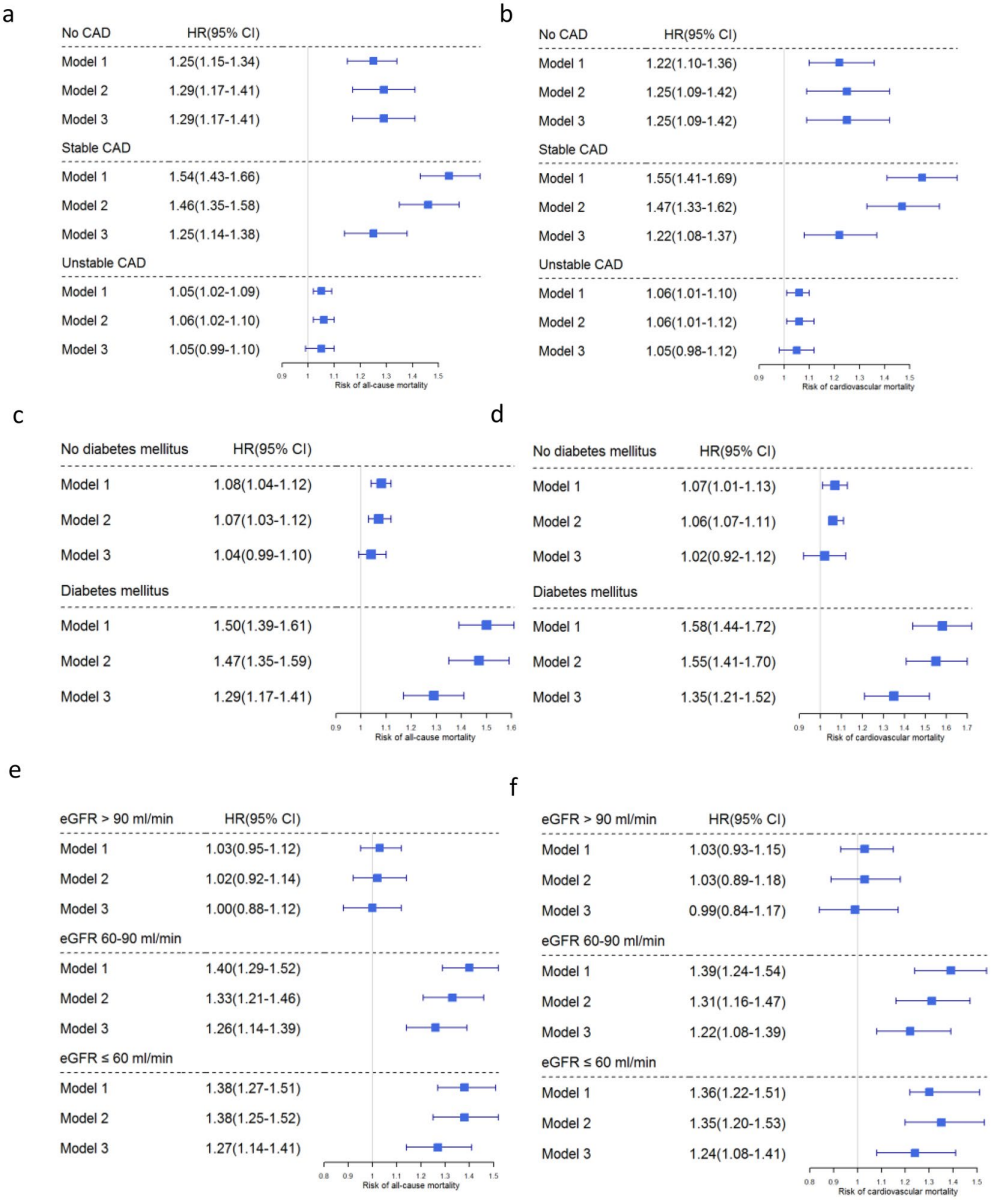
Subgroup analysis on predictors of a. all-cause mortality and b. cardiovascular mortality in patients with coronary artery disease (CAD), c. all-cause mortality and d. cardiovascular mortality in patients with diabetes mellitus, and e. all-cause mortality and f. cardiovascular mortality in chronic kidney disease patients (CKD). The hazard ratios show the risk per increase of one standard deviation.

In patients with stable CAD, all-cause mortality was best predicted by models 1 and 2, meaning that suPAR is a predictive biomarker after adjustment for age and sex (model 1) and further adjustment for traditional risk factors. Adjustment for inflammation markers, NT-proBNP and eGFR, however, did not further enhance prediction of all-cause and CV mortality (Fig. 3a,b). In patients with acute coronary syndrome (unstable CAD), suPAR only marginally improved the prediction of all-cause or CV mortality risk. The subgroup without angiographic CAD was too small and had too few events to derive meaningful estimates.

#### Patients with diabetes

In patients with diabetes mellitus all models predicted all-cause and CV morality (Fig. 3c,d). suPAR remains as predictive biomarker even after adjustment for multiple confounders.

#### Patients with chronic kidney disease

Among the LURIC patients studied, 13.5% (398 of 2940) were at CKD stage 3 and higher at baseline. Median suPAR level of these CKD patients was 4520 (3528–5.898) pg/mL. Plasma suPAR levels increased with CKD stages (pg/mL; stage 1 2590 (1950–3350), stage 2 3070 (2310–3943), stage 3 4420 (3415–5735), stage 4 5370 (4300–7620), stage 5 6845 (5400–11198)). In the multivariate regression analysis eGFR was one of the most important independent “predictors” of suPAR (Table 3). Addition of suPAR to a model with traditional risk factors also improved significantly risk stratification for all-cause and CV mortality in the CKD subgroup (Table 6, Fig. 3e,f). Including heart failure and peripheral artery disease to the model confirmed the predictive value of suPAR (Table S2).

## Discussion

This comprehensive *post hoc* analysis of the LURIC cohort represents one of the largest clinical studies examining the predictive value of suPAR. SuPAR was a strong predictor of all-cause and especially CV death over a period of ten years in persons undergoing coronary angiography. This association was not only independent of age, gender and other traditional CV risk factors, but also independent of strongly prognostic cardiac biomarkers as NT-proBNP and inflammation markers as hs-CRP and IL-6. The risk of all-cause and CV mortality increased gradually in parallel to the suPAR concentration with a very rapid risk increase until a suPAR level of 4000 pg/mL. Such an increase of risk with rising suPAR levels could be shown for all different components of the endpoint of CV death and, beyond this, for infections leading to death. Addition of suPAR to a model including traditional risk factors, leads to an improved risk prediction in the entire LURIC population as well as in the CAD and CKD subgroups. The latter shows that elevated suPAR levels were not merely a reflection of decreased renal function. Rather, suPAR may actively affect the prognosis of CKD patients independent of traditional risk factors and risk factors typical for CKD patients such as increased NT-proBNP and hsCRP.

In an analysis of the Emory Cardiovascular Biobank high levels of plasma suPAR were associated with the presence and severity of CAD and were independent predictors of death and myocardial infarction in patients with suspected or known CAD^12^. The Danish MONICA study has shown in a cohort of 2602 patients that suPAR is associated with all-cause mortality and CV disease^7^. Further, the addition of suPAR improved CV risk prediction beyond the Framingham Risk Score^6^. In another small Danish single center study, suPAR concentrations measured in 449 chest pain patients was predictive for death during a median follow-up of 5.7years independent of age, sex, smoking, and comorbidities^13^. In the LURIC study, suPAR levels contributed significantly to predict not only all-cause and the summary of CV mortality but also all different causes of CV death as sudden cardiac death, fatal myocardial infarction, congestive heart failure as well as fatal stroke in the LURIC study.

The present investigation is the first evaluation of suPAR in a large prospective clinical cohort of about 3000 Caucasians undergoing coronary angiography with a long-term follow-up. Another advantage of the LURIC study is that it includes a comprehensively characterized patient cohort in whom also strongly predictive, emerging biomarkers were available. This allowed us to prove that the predictive value of suPAR for the endpoints all-cause and CV mortality was independent of NT-proBNP, IL-6 and hsCRP.

NT-proBNP is a well-known diagnostic biomarker for heart failure and CV mortality and a predictor of CV outcome in the general population and in patients with varying underlying disease^14,15^. In the LURIC study, NT-proBNP levels increased significantly with each suPAR quartile and the multivariate regression analysis revealed NT-proBNP as independently correlated with suPAR. Yet, as to CV mortality, the HR was as high as 2.75 (95% CI 2.03–3.71) in the highest compared to the lowest suPAR quartile after adjusting for traditional risk factors along with renal function, inflammation markers, and NT-proBNP.

SuPAR appears to be involved in pathophysiological pathways linked to atherosclerosis different from inflammatory processes^4,16^. The finding that suPAR might be stronger associated with atherosclerosis than CRP has become apparent from the Danish Risk Score study^17^. Statin treatment exhibits anti-inflammatory effects^18^. In the present study, percentages of patients with statins were similar in all four suPAR quartiles and LDL-C did not significantly increase with suPAR quartiles. In support of this, suPAR outperformed not only the highly predictive marker NT-proBNP, but also at the same time the strong inflammation markers hsCRP and IL-6.

suPAR as significant predictor of incident mortality and morbidity in patients with suspected or established CAD was detected by Eapen *et al*. in the Emory database^12^. In the LURIC study, subgroup analysis of patients with stable CAD, the HR estimates decreased when adjusted for inflammation, NT-proBNP and eGFR. Similarly, especially subgroups prone to CV events as patients with impaired renal function or diabetes mellitus revealed a decrease of HR estimates when adjusting for these confounders. These results are in line with a *post hoc* analysis of the 4D study on the association of suPAR in diabetic hemodialysis patients and all-cause or CV death^19^. In patients with acute coronary syndrome, suPAR only marginally improved the prediction of fatal outcomes, but this slight improvement of prediction was robust after adjusting for CV risk factors. In a small earlier study of patients with ST-elevation myocardial infarction, suPAR did not change during the acute phase of the disease^20^. Similarly, coronary artery bypass surgery did not affect suPAR levels^21^, together also suggesting that suPAR is not acting as a classical acute phase reactant.

In patients with impaired renal function, suPAR was a strong predictor for all-cause and CV mortality. In this subgroup, suPAR retained its predictive value even after adjustment for typical cardiac risk factors in CKD. The association of suPAR with outcomes in renal failure has been shown in the 4D study and in a small earlier report of 476 patients with mild-moderate CKD in which suPAR correlated with mortality and incident CV events^19,22^. All these results show that suPAR remains predictive for CV outcome even if renal function declines. This is of special importance since suPAR itself has been suggested to be predictive of incident renal CKD and may have a pathogenic component in renal disease^11,23–26^. Hayek *et al*. demonstrated that suPAR was linked to incident CKD and an accelerated decline in eGFR in patients with pre-existing CKD. In the present LURIC population, prevalence of CKD increased significantly with suPAR quartiles either after adjustment for age and sex as well as after adjustment for traditional risk factors.

Among the limitations of the present study might be the study design which was a *post hoc* analysis within a cohort of inhabitants of a geographic area in Germany. However, all patients undergoing coronary angiography in this area independent of co-morbidity were enrolled. Therefore, at least a generalization to Caucasian patients is feasible. Despite of adjustments for multiple well-known confounders additional confounding is possible. Analyses and results of subgroups as CKD patients or patients with acute myocardial infarction are weaker compared to the total patient cohort due to the smaller sample size. Future therapeutic options relating to suPAR could also not be addressed.

This comprehensive evaluation on the predictive value of suPAR for all-cause and CV mortality in a *post hoc* analysis of the large LURIC study confirmed suPAR as an extraordinarily strong predictor of all-cause and especially CV mortality over a period of ten years in Caucasian persons undergoing coronary angiography, independent of cardiac and kidney function and markers of systemic inflammation. In addition, this effect could also be seen in patients with mild and moderate CKD. Altogether, the present analysis contributes to our understanding evaluation of suPAR as a risk marker. Further research is needed to elucidate a potential underlying pathophysiology for suPAR and cardiovascular disease.

## Methods

### Study design and participants

In the present *post hoc* analysis, suPAR was measured in baseline blood samples from patients participating in the LURIC study. The LURIC study is a prospective cohort study on 3316 Caucasians referred to coronary angiography between 1997 and 2000 at the Ludwigshafen Heart Center in German. Indications for angiography were clinical symptoms as chest pain or a positive non-invasive stress test suggesting myocardial ischemia. Individual suffering from acute illness other than acute coronary syndromes, chronic disease not primarily CV and history of malignancy within the last 5 years as well as subjects not able to understand the purpose of the study were excluded^27^.

The study was approved by the local ethics committee (“Landesärztekammer Rheinland-Pfalz”, no. 1997–203) and conducted in accordance with the principles of the Declaration of Helsinki. Informed written consent was obtained from all participants.

### Clinical data collection

Standardized questionnaires and collection of clinical data from the patients´ charts were obtained from all enrolled subjects.

The presence of a visible luminal narrowing (>20% stenosis) in at least one of 15 coronary segments was used to define coronary artery disease (CAD) according to the classification of the American Heart Association. Diabetes mellitus was defined according to 2010 guidelines of the American Diabetes Association as increased fasting (≥126 mg/dl) and/or post-challenge (2 h after the 75 g glucose load >200 mg/dl) glucose and/or elevated glycated hemoglobin (>6.5%) and/or history of diabetes. Hypertension was defined as a systolic and/or diastolic blood pressure ≥140 and/or ≥90 mm Hg or a history of hypertension. The glomerular filtration rate was estimated by using the 2012 CKD-EPI eGFRcreat-cys equation^28^ and the patients were stratified into categories of their eGFR according to the Kidney Disease: Improving Global Outcomes (KDIGO) guidelines^29^.

Information on vital status was obtained from local registries. Death certificates, medical records of local hospitals, and autopsy data were reviewed independently by two experienced clinicians who were blinded to patient characteristics and who classified the causes of death. CV mortality was defined as death due to fatal myocardial infarction, sudden cardiac death, death after CV intervention, stroke and other causes of death due to CV diseases.

### Laboratory measurements

Fasting blood samples were obtained by venipuncture in the early morning in a standardized procedure^27^. Blood glucose, cholesterol, triglycerides, low- and high-density lipoprotein cholesterol (LDL-C, HDL-C), fibrinogen, calcium, phosphate, gamma glutamyltransferase (yGT), albumin, glycosylated hemoglobin (HbA1c), blood account were determined by common laboratory assays. Intact parathormone (iPTH) was assessed using an ElectroChemiLuminescence Immunoassay (ECLIA) on an Elecsys 2010 (Roche Diagnostics, Mannheim, Germany). High-sensitivity C-reactive protein (hsCRP) was measured on a BN II analyzer by nephrelometry (Dade Behring, Marburg, Germany), interleukin 6 (IL-6) was measured by enzyme-linked immunosorbent assay (high sensitivity, Quantikine kit; R&D Systems, Wiesbaden, Germany). N-terminal pro brain natriuretic peptide (NT-pro-BNP) was measured by electro-chemiluminescence on an Elecsys 2010 (Roche Diagnostics).

### suPAR measurements

Plasma suPAR levels were determined in blood samples taken at baseline by ELISA (suPARknostic kit; ViroGates, Copenhagen, Denmark), with a lower detection limit of 100 pg/mL. Intra- and inter-assay variation was 2.75% and 9.17%, respectively. These blood samples had been stored for a median of 18 years in controlled −80 °C refrigerators. SuPAR measurements have been assessed to be stable in long-term storage and are minimally affected by repeated freezing and thawing cycles. Technicians measuring suPAR were blinded to clinical outcome data.

### Statistical analyses

All continuous variables were checked for normality and variables showing a skewed distribution were logarithmically transformed. Continuous variables were compared between groups by ANOVA. Associations between categorical variables were examined by chi-square testing. suPAR was examined as quartiles or as standardized, Z-transformed values. The Z-score is calculated by subtracting the sample mean from the individual raw values and then dividing the difference by the sample standard deviation. To examine the relationship with mortality we calculated hazard ratios (HR) and 95% confidence intervals (95% CI) using the Cox proportional hazards model. Multivariable adjustment was carried out as indicated. The proportional hazard assumption was checked by examination of scaled Schoenfeld residuals. HR plots were drawn using the R-package ‘rms’ (v5.1–1) with suPAR modeled as restricted cubic spline with three knots. For the calculation of Harrells C we first calculated the linear predictors of the respective Cox regression models and used these as input for the rcorrcens function as implemented in the R-package Hmisc (v 4.1-1). ROC curves based on binary logistic regression models were calculated and compared using the method of Delong as implemented in the R package ‘pROC’ (v1.8). IBM SPSS Statistics v. 22.0 (IBM Corporation) and R statistical software v. 3.4.0 (http://www.r-project.org) was used for all analyses.

## Electronic supplementary material

Supplementary Information
