# Supplementary material for: Soluble urokinase plasminogen activation receptor and long-term outcomes in persons undergoing coronary angiography
Source: Sci Rep. 2019 Jan 24;9:475. doi: 10.1038/s41598-018-36960-6 (PMC6346054; doi:10.1038/s41598-018-36960-6)
Supplement: Supplementary file 1 — Supplementary Information [file 41598_2018_36960_MOESM1_ESM.docx]

Supplementary Material

**Soluble urokinase plasminogen activation receptor and long-term outcomes in persons undergoing coronary angiography**

Claudia Sommerer, MD^1^, Martin Zeier, MD^1^, Christian Morath, MD^1^, Jochen Reiser, MD, PhD^2^, Hubert Scharnagl, PhD^3^, Tatjana Stojakovic, MD^3^, Graciela E Delgado, Msc^4^, Winfried März, MD^3,4,5^, Marcus E Kleber, PhD^4^

1 Department of Nephrology, University of Heidelberg, Heidelberg, Germany
2 Department of Medicine, Rush University Medical Center, 1735 West Congress Parkway, Suite 1004, Chicago, IL 60612, USA

3 Clinical Institute of Medical and Chemical Laboratory Diagnostics, Medical University of Graz, Graz, Austria
4 Vth Department of Medicine, Medical Faculty Mannheim, University of Heidelberg, Germany

5 Synlab Academy, Synlab Holding Deutschland GmbH, Mannheim, Germany

**Table S1.**

Risk (hazard ratio and 95% confidence interval) of death due to fatal infection and fatal cancer stratified by quartiles of soluble urokinase plasminogen activator receptor (suPAR) concentration (pg/mL) at baseline (study population n=2940).

|  | Model 1: Demographics | | Model 2:  Demographics  + traditional CV risk factors | | Model 3: Demographics  + traditional CV risk factors  + specific CV risk factors | |
| --- | --- | --- | --- | --- | --- | --- |
|  | HR (95% CI) | p | HR (95% CI) | p | HR (95% CI) | p |
| *Death due to fatal infection (72 events)* | | |  |  |  |  |
| Quartile 1 | 1  (reference) |  | 1  (reference) |  | 1  (reference) |  |
| Quartile 2 | 1.11  (0.39-3.22) | 0.841 | 1.08  (0.37-3.13) | 0.884 | 0.98  (0.34-2.85) | 0.974 |
| Quartile 3 | 2.55  (1.01-6.44) | 0.048 | 2.39  (0.94-6.09) | 0.068 | 1.75  (0.68-4.51) | 0.248 |
| Quartile 4 | 5.65  (2.35-13.58) | <0.001 | 5.19  (2.11-12.77) | <0.001 | 2.51  (0.97-6.50) | 0.058 |
| *Death due to fatal cancer (128 events)* | | |  |  |  |  |
| Quartile 1 | 1  (reference) |  | 1  (reference) |  | 1  (reference) |  |
| Quartile 2 | 0.68  (0.40-1.16) | 0.161 | 0.62 (0.36-1.05) | 0.076 | 0.57 (0.33-0.99) | 0.045 |
| Quartile 3 | 0.99 (0.61-1.62) | 0.977 | 0.85 (0.52-1.40) | 0.527 | 0.85 (0.51-1.41) | 0.520 |
| Quartile 4 | 1.26 (0.78-2.04) | 0.352 | 0.98 (0.59-1.63) | 0.931 | 0.95 (0.55-1.63) | 0.843 |

**Table S2**

Prediction of all-cause mortality and cardiovascular mortality with traditional risk factors (age, sex, body-mass index, LDL-C, HDL-C, smoking, hypertension, diabetes mellitus) including heart failure and peripheral artery disease with and without soluble urokinase plasminogen activator receptor (suPAR) concentration.

| All-cause mortality |  |  |  |
| --- | --- | --- | --- |
|  | Harrells C | AUC (95% CI) | P |
| *All participants (n=2940)* | |  |  |
| Base | 0.752 | 0.796 (0.778-0.813) |  |
| Base + suPAR | 0.757 | 0.806 (0.789-0.823) | <0.001* |
| Base + hsCRP | 0.753 | 0.796 (0.778-0.813) | 0.624* |
| Base + IL-6 | 0.757 | 0.800 (0.782-0.817) | 0.005* |
| Base + NT-proBNP | 0.758 | 0.803 (0.786-0.820) | <0.001* |
| Base + NT-proBNP + suPAR | 0.762 | 0.809 (0.793-0.826) | <0.001** |
|  |  |  |  |
| *Only CAD patients (n=2302)* | |  |  |
| Base | 0.739 | 0.784 (0.764-0.804) |  |
| Base + suPAR | 0.743 | 0.794 (0.774-0.813) | <0.001* |
| Base + hsCRP | 0.739 | 0.784 (0.764-0.804) | 0.949* |
| Base + IL-6 | 0.743 | 0.787 (0.768-0.807) | 0.034* |
| Base + NT-proBNP | 0.745 | 0.791 (0.771-0.810) | <0.001* |
| Base + NT-proBNP + suPAR | 0.748 | 0.796 (0.777-0.815) | <0.001** |
|  |  |  |  |
| *Only CKD patients (n=398)* | | |  |
| Base | 0.656 | 0.729 (0.678-0.780) |  |
| Base + suPAR | 0.682 | 0.752 (0.702-0.801) | 0.076* |
| Base + hsCRP | 0.656 | 0.731 (0.680-0.783) | 0.594* |
| Base + IL-6 | 0.659 | 0.730 (0.679-0.782) | 0.552* |
| Base + NT-proBNP | 0.668 | 0.735 (0.684-0.785) | 0.331* |
| Base + NT-proBNP + suPAR | 0.687 | 0.752 (0.702-0.801) | 0.145** |

Cardiovascular mortality

|  | Harrells C | AUC (95% CI) | p |
| --- | --- | --- | --- |
| *All participants (n=2922)* | |  |  |
| Base | 0.772 | 0.783 (0.763-0.803) |  |
| Base + suPAR | 0.775 | 0.788 (0.768-0.808) | <0.001* |
| Base + hsCRP | 0.772 | 0.783 (0.763-0.803) | 0.958 |
| Base + IL-6 | 0.776 | 0.786 (0.766-0.806) | 0.028* |
| Base + NT-proBNP | 0.779 | 0.790 (0.770-0.810) | <0.001* |
| Base + NT-proBNP + suPAR | 0.782 | 0.792 (0.773-0.812) | 0.013** |
|  |  |  |  |
| *Only CAD patients (n=2284)* | |  |  |
| Base | 0.756 | 0.766 (0.743-0.789) |  |
| Base + suPAR | 0.760 | 0.771 (0.748-0.794) | 0.002 |
| Base + hsCRP | 0.756 | 0.766 (0.743-0.789) | 0.902* |
| Base + IL-6 | 0.759 | 0.769 (0.746-0.792) | 0.105* |
| Base + NT-proBNP | 0.762 | 0.772 (0.750-0.795) | 0.002* |
| Base + NT-proBNP + suPAR | 0.765 | 0.775 (0.752-0.797) | 0.026** |
|  |  |  |  |
| *Only CKD patients (n=393)* | | |  |
| Base | 0.671 | 0.675 (0.622-0.728) |  |
| Base + suPAR | 0.693 | 0.681 (0.628-0.734) | 0.298* |
| Base + hsCRP | 0.670 | 0.677 (0.624-0.730) | 0.583* |
| Base + IL-6 | 0.673 | 0.674 (0.621-0.727) | 0.754* |
| Base + NT-proBNP | 0.681 | 0.675 (0.622-0.728) | 0.959 |
| Base + NT-proBNP + suPAR | 0.694 | 0.681 (0.629-0.734) | 0.355** |

* P versus base model; ** P versus Base + NT-proBNP

AUC, area under the curve; CAD, coronary artery disease; CI, confidence interval; CKD, chronic kidney disease; p significance; suPAR, soluble urokinase plasminogen activator receptor
